# Spinal ultrasound imaging implied the vascular dynamics in chronic and acute pain

**DOI:** 10.1016/j.mex.2025.103273

**Published:** 2025-03-15

**Authors:** Yingzhuo Ding, Chunxia Yu, Qingqing Chi, Mengjiao Deng, Dongxia Duan, Jinbao Wei, Yufei Xi, Qin Li, Le Ma

**Affiliations:** a Shanghai Eye Diseases Prevention &Treatment Center/ Shanghai Eye Hospital, School of Medicine, Tongji University, National Clinical Research Center for Eye Diseases, Shanghai Engineering Research Center of Precise Diagnosis and Treatment of Eye Diseases, NO.1440 Hongqiao Road, 200336 Shanghai, China; b Shanghai Pulmonary Hospital, Tongji University of Medicine, 507 Zhengmin Road, 200433 Shanghai, China; c Shanghai Mental Health Center, Shanghai Jiao Tong University School of Medicine, 201108 Shanghai, China; d Department of Pharmacy, Xiamen Haicang Hospital, 361026 Xiamen, China; e Department of Clinical Pharmacy, Shanghai General Hospital, Shanghai Jiao Tong University School of Medicine, No.100 Haining Road, 200080 Shanghai, PR China

**Keywords:** Pain, Neuropathic pain, Formalin pain, Spinal functional ultrasound imaging, Gabapentin, spinal functional ultrasound imaging

## Abstract

Neurovascular coupling links local neural activity to cerebral blood flow changes, crucial for pain transduction. In this undertaking, we present an advanced spinal functional ultrasound imaging that exhibits precise observation capabilities of the influence of analgesic medications on spinal cord blood flow, encompassing both acute and chronic pain scenarios. Utilizing gabapentin as the primary analgesic agent, we have observed that intrathecal administration of gabapentin notably augments the blood flow intensity in neuropathic rats while exerting minimal influence on the blood flow of the sham rats. Furthermore, subcutaneous formalin injection increases spinal cord blood flow, but gabapentin pretreatment prevents those effects. These findings demonstrated the analgesic effect of gabapentin exhibits a synergistic interaction with the intensity of blood flow around neurons, which is crucial for understanding the mechanisms underlying the occurrence of pain. In this article, we show:

• A simple method to build the spinal cord ligation-induced chronic pain model.

• A generic way to build the formalin-induced acute pain model.

• An optimized functional ultrasound imaging technique in chronic and acute pain.

## Graphical abstract

**Figure:**
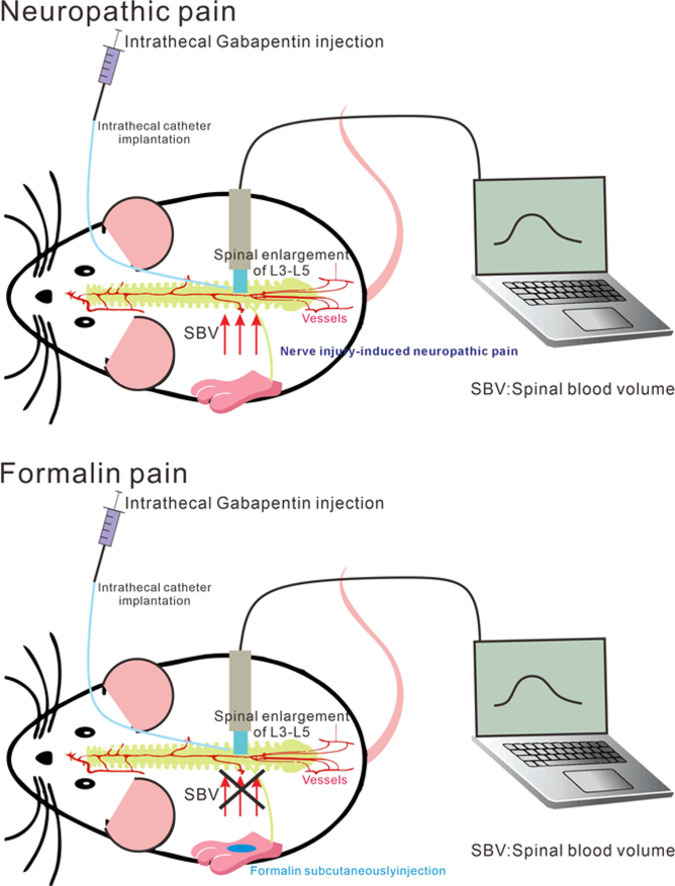

Specifications table

**Table:** 

Subject area:	Pharmacology, Toxicology and Pharmaceutical Science
More specific subject area:	Pain
Name of your method:	spinal functional ultrasound imaging
Name and reference of original method:	Ma L, Sun Y, Liu B, Shi Y, Luo C, Cheng Y, Wang W, Fang Y, Huang L, Ali U, Zhang J, Chen J, and Ju P. Andrographolide exhibits antinociceptive effects in neuropathic rats via inhibiting class Ⅱ MHC associated response and regulating synaptic plasticity. Phytomedicine. 2024; 132: 155823.
Resource availability:	The whole ultrasonic probe (14 mm, f = 15 MHz, 128 elements, Vermon, Tours, France).
	The ultrasonic ultrafast scanner (Verasonics, Kirkland, WA: 128 channels, 62.5 MHz sampling rate).
	Neuroscan live acquisition software (Iconeus, Paris, France; and Inserm Accelerator of Technological Research in Biomedical Ultrasound, Paris, France).

## Background

The brain, as the most complex and metabolically active organ in the human body, relies heavily on a continuous and stable supply of glucose and oxygen for its functions and metabolic activities. The effective delivery of these two crucial substrates is directly constrained by the dynamic regulation of cerebral blood flow. In this process, the mechanism of neurovascular coupling plays a central role, ensuring that when neuronal activity increases, blood flow to the brain can rapidly and precisely rise to meet its metabolic demands. Notably, even slight fluctuations in neuronal activity can trigger corresponding adjustments in local blood flow within seconds, establishing a solid foundation for the rapidity and accuracy of neural electrophysiological activity. However, when the neurovascular coupling function is impaired, this finely tuned regulatory mechanism can be disrupted, leading to close associations with various central nervous system disorders, especially in neuropathic pain. In these conditions, the neurovascular decoupling phenomenon is viewed as a critical pathophysiological mechanism and serves as a potential driving force for pain progression and deterioration. Therefore, employing non-invasive techniques to investigate changes in neurovascular coupling under pain states can enhance our understanding of its pathophysiological processes and may reveal key diagnostic indicators. Moreover, by continuously monitoring the status of neurovascular coupling, abnormal changes in cerebral blood flow in the early stages of pain can be detected, providing valuable insights for early diagnosis and timely intervention. Notably, the neurovascular coupling mechanism also opens new perspectives and approaches for treating pain. For instance, by optimizing the neurovascular coupling process, we can effectively increase cerebral blood perfusion, alleviating symptoms of hypersensitivity and improving treatment outcomes. During treatment, the ongoing monitoring of neurovascular coupling changes can provide scientific evidence for evaluating pain treatment efficacy and adjusting strategies, thereby enhancing therapeutic outcomes and the quality of life for patients with neuropathic pain. Consequently, future efforts must delve deeper into the structure and function of the spinal dorsal horn to unveil its precise role in pain processing, paving the way for novel pain management strategies and methodologies.

Spinal functional ultrasound (fUS) imaging is a leading-edge and versatile neuroimaging method. It has proven its mettle in delivering high-precision imaging and quantifying cerebral blood volume in larger animals while maintaining superb spatial and temporal resolution [1]. Considering the interconnectivity between nervous and vascular systems, a discernible hemodynamic correlation exists between regional blood flow and adjacent neuronal activity.

This attribute positions fUS imaging as a highly promising tool in various applications, notably spinal cord blood flow analysis and pain signal evaluation. The primary aim of our investigation is to explore the hemodynamic responses in chronic neuropathic pain and acute formalin-induced pain. More importantly, we intend to conduct a rigorous assessment of the specific hemodynamic effects triggered by intrathecal administration of gabapentin, a prevalent analgesic in clinical settings. This endeavor is poised to facilitate nuanced blood flow monitoring across diverse pain scenarios.

## Method details

### Preparation of artificial cerebrospinal fluid (ACSF)

To prepare sterile artificial cerebrospinal fluid, use the following formula (in mM): 3 KCl, 125 NaCl, 26 NaHCO_3_, 1.25 NaH_2_PO_4_, 1 MgCl_2_, 10 D-glucose, and 2 CaCl_2_ (pH adjusted to 7.3) [2].

### Animals

The naïve male Wistar rats were purchased from Shanghai Leigen Biotechnology Co., Ltd and used in this study in a conventional controlled environment. All the procedures were conducted in accordance with the guidelines approved by the Committee for Animal Care and Use of Laboratory Animals of Shanghai Jiao Tong University (approval No. 10902), followed the National Institutes of Health Guide for the Care and Use of Laboratory Animals, and in compliance with the 3Rs framework and ARRIVE guidelines recommended.

### Spinal nerve ligations

Animals were produced by left L5 and L6 ligation under inhaled isoflurane. Briefly, the skin was incised, and the left paravertebral region was exposed. The spinal nerve L5, relatively parallel to the spinal cord, was isolated to obtain the spinal nerve according to the anatomical structure. The L6, relatively perpendicular to the spinal column, was also dissected. Ligations were made to L5 and L6 with a No. 6-0 silk ligature. All the surgeries were carried out on rats under deep anesthesia, and the spinal nerves of the right side of the rat were not operated upon. Rat developed neuropathic pain 14 days post-surgery, and a paw withdrawal threshold < 8 g were chosen for further experiment according to previous study [3,4].

### Paw withdrawal threshold test

In the four days preceding the commencement of the behavioral study, arrangements were meticulously made for the rats to undergo a comprehensive environmental acclimation process, along with handling procedures. During this carefully planned period, the rats were kept there for a minimum of 30 min. This gradual exposure aimed to facilitate their adjustment to the unfamiliar laboratory environment. Throughout the entire handling process, the experimenters took utmost precaution, wearing protective gloves to prioritize the safety of the rats and themselves. On the first day of the acclimation training, the experimenters delicately inserted their hands into the rats' housing containers. They carefully stroked their back skin twice, ensuring each session lasted approximately a minute. As the second day progressed, the rats were cautiously placed onto a metal grate, and the experimenters continued to gently stroke them in alignment with their fur for 1-2 min per session. This meticulous handling routine was sustained until the end of the experiment to familiarize the rats with the experimenters through scent recognition and repeated gentle handling. These measures were implemented to mitigate any potential stress or adverse emotional responses that could arise from the experimental manipulations during the actual study, ultimately ensuring the experimental results’ integrity, accuracy, and reliability. Following the conclusion of the handling procedure, the rats were carefully transferred to a transparent plexiglass chamber positioned on a metal mesh platform, elevated approximately 40 cm above the laboratory bench. After 30 min of acclimation to the new environment, the rats' mechanical pain threshold was measured using a Von Frey filament (2450 CE electronic Von Frey) equipped with a 15-gauge fiber. During the measurement phase, vertical upward stimulation was applied to the intersection of the third and fourth toes of the rats' left hind paws. The minimum stimulus intensity required to elicit an avoidance behavior, such as paw withdrawal or lifting, was observed, and meticulously recorded. This threshold served as a quantifiable metric, the mechanical pain threshold, and was subsequently utilized to assess the rats' sensitivity to painful stimuli. To ensure data reliability, three consecutive measurements of each rat's threshold were conducted at designated time points, and the average value was calculated to derive the threshold. A consistent interval of 3 min was maintained between each measurement to allow for adequate recovery time and ensure accuracy according to previous study [2,5].

### Formalin test

Formalin solution was prepared at 5 % in normal saline from a formalin stock (Nanchang, Jiangxi, China). Rats were placed individually into Plexiglas chambers and allowed to habituate in the testing environment for at least 30 min. Each rat was injected subcutaneously with 50 µL of 5 % formalin dorsally on the left hind paw and immediately placed individually in a glass observation chamber (15 × 14.5 × 14.5 cm), which was embedded at a 45-degree angle mirror for 60 min. The pain-related behaviors, which included licking, flinching, and biting of the injected paw, were observed at 10 min intervals. The time the rat exhibited these pain behaviors during the first phase (0-10 min) and the second phase (10-60 min) was recorded for statistical comparisons. In summary, formaldehyde induces acute inflammatory pain through a combination of direct chemical stimulation of nociceptors (Phase I) and the subsequent inflammatory reaction, which leads to peripheral and central sensitization (Phase II). In the investigation of the effects of the gabapentin,gabapentin was purchased from Merck Millipore (Sigma-Aldrich, Catalog No. Y0001280, CAS No. 60142-96-3) and dissolved in ACSF for intrathecal injection. According to previous study [6,7], gabapentin (or ACSF) was injected intrathecally 45 min before formalin administration. Gabapentin (or ACSF) and formalin were administered using microsyringes and U-40 insulin syringe with a 29-gauge needle, respectively.

### Intrathecal catheter implantation

With the rat under isoflurane anesthesia, a 1.0 cm long incision was made along the dorsal skin at the iliac position. We implanted the 22 cm polyethylene catheter (PE-10 catheter) with ID: 0.30 mm and OD: 0.55 mm into the subarachnoid space of the spine with a 9-gauge needle. The tip of the catheter was close to where the last rib was perpendicular to the spine. At this point, a typical tail flick could be observed. The catheter was fixed near the iliac bone and buried beneath the skin. When the animal was ready for ultrasound recording, the catheter can be located by exposing the lumbar enlargement area iliac bone. An appropriate amount of penicillin was given to the skin opening to prevent surgical infection, and the wound was sutured. The rats were transferred to a heating pad to recover consciousness and placed in a single-cage housing. Two days after recovery, 10 µl of 4 % lidocaine dissolved in ACSF was injected from the catheter with a 15 µL microsyringe, followed by a 15 µL ACSF flush, and the bilateral hind feet were lim and weak. There was no occurrence of motor impairments such as paralysis and claudication after the metabolization of lidocaine. The simultaneous fulfillment of the above two conditions indicated that the intrathecal catheter was at the correct position and could be used for subsequent experiments, according to a previous study [8].

### Functional ultrasound imaging

The rat was deeply anesthetized with isoflurane (R510-22, RWD China, 4–5 % induction, 1–2 % maintenance) in O_2_ by inhalation and in the prone position. A window was made between the 12th thoracic vertebra and the second lumbar vertebra to perform a laminectomy. The muscles surrounding the spinal cord of rats were gently removed with clippers and forceps to expose the lumbar vertebrae. The lumbar expansion was then carefully thinned until the spinal cord was exposed. Antibiotics were added to the wound, and the wound was sutured. Finally, 8.0 × 10^5^ units of penicillin were injected intramuscularly to prevent infection, and the wound was sutured. Functional ultrasound imaging was recorded one week after surgery.

Once the laminectomy was performed, the rat was placed on a customized “spinal cord” stereotaxic frame, where the lumbar rachis was tightly clipped. The incision was opened to expose the spinal cord. An ultrasound-specific separator membrane was applied to the exposed tissue portion to isolate the imaging gel from the tissue and filled with ultrasound gel. The whole ultrasonic probe (14 mm, f = 15 MHz, 128 elements, Vermon, Tours, France) was positioned in a sagittal plane and above the window using a 3-axis motor system where the probe was fixed. The probe, which was connected to an ultrasonic ultrafast scanner (Verasonics, Kirkland, WA: 128 channels, 62.5 MHz sampling rate), was controlled by Neuroscan live acquisition software (Iconeus, Paris, France; and Inserm Accelerator of Technological Research in Biomedical Ultrasound, Paris, France). The rat's body temperature was maintained at 37 ˚C with a heating pad and an intrarectal probe, and heart and respiratory frequencies were monitored during the surgery and imaging process. Each imaging session lasted from 1 to 2 h.

### Data analysis

This study used GraphPad Prism 7.0 for statistical analysis, presenting all data as mean ± SEM. To ensure data normality, logarithmic transformations preceded the Shapiro-Wilk test. Gaussian-distributed data were analyzed via an unpaired two-tailed t-test, with statistical significance set at p < 0.05. Relevant F-values, degrees of freedom, and P-values are provided. All graphics were optimized using CorelDraw 2020.

### Method validation

**Fig. 1:**
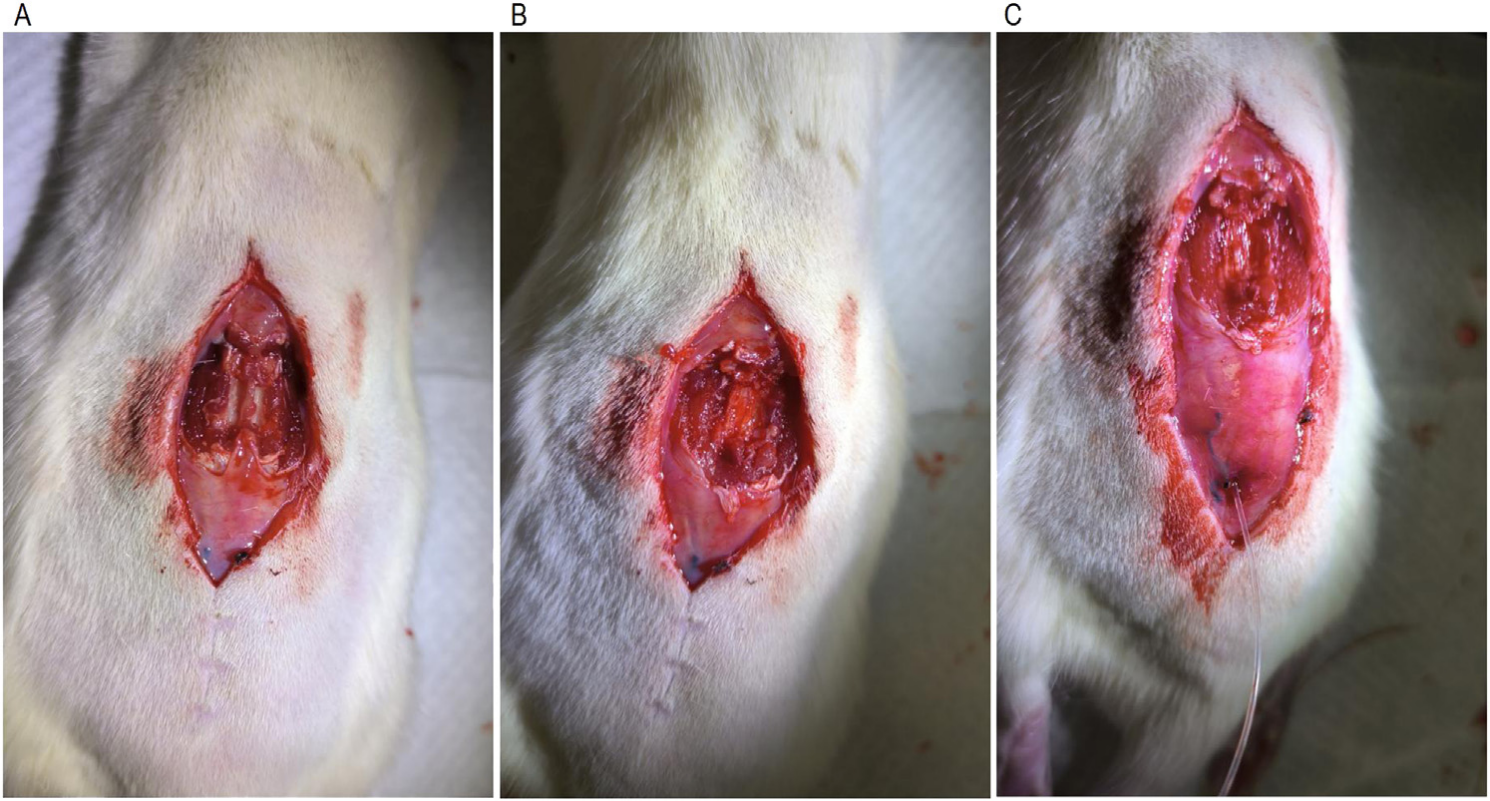
Spinal laminectomy and intrathecal catheterization. A. Window for L3-L5, B. Spinal laminectomy of spinal enlargement, C. Intrathecal catheterization targeting spinal enlargement in rats.

Following one week of recovery from SNL surgery, male rats underwent a laminectomy procedure. Rats were anesthetized using isoflurane (R510-22, sourced from RWD China) at an induction concentration of 4-5 % and maintained at 1-2 % via inhalation of O_2_. They were then positioned in a stereotaxic frame for surgical precision. Guided by anatomical landmarks, an incision was made directly above the L3-L5 vertebrae. The skin was dissected parallel to the spine, revealing the spinous processes. A surgical drill was used to thin the sides of the spinous processes, which were then grasped with hemostatic forceps and elevated to expose the spinal cord. Any excess bone was trimmed using fine scissors, ensuring maximal exposure of the spinal cord tissue while carefully avoiding blood vessel damage (shown in Fig. 1A and B). Next, a subarachnoid catheterization was performed by inserting a catheter tip containing artificial cerebrospinal fluid into the exposed spinal cord area (shown in Fig. 1C). The top tube of the catheter inserted through the sheath is precisely positioned at the exposed area of the spinal L3-L5. This ensured precise drug delivery to the enlarged spinal cord segment, facilitating the investigation of drug effects on functional connectivity and blood flow in specific regions. The catheter was then secured, the incision sutured, and antibiotics administered to prevent post-surgical infection. Rats were placed on a 37 ˚C heating pad until they regained consciousness before being returned to their cages for a one-week recovery period. Before experimentation, animals were allowed to acclimate to the experimental environment.

### Recordings in neuropathic rats

Neuropathic rats were assessed by paw withdrawal thresholds < 8 g. Rats were anesthetized with isoflurane and positioned in a customized operating frame, exposing the spinal cord area. To maintain the stability of the spinal cord tissue during extended recordings, specialized clamps were used to fix the spinal cord on both sides of the exposed area. This prevented respiratory movements from affecting the spinal cord, ensuring a completely prone position for accurate recordings. After allowing the animals to acclimate for 30 min, the spinal cord tissue and previously placed catheter were re-exposed.

**Fig. 2:**
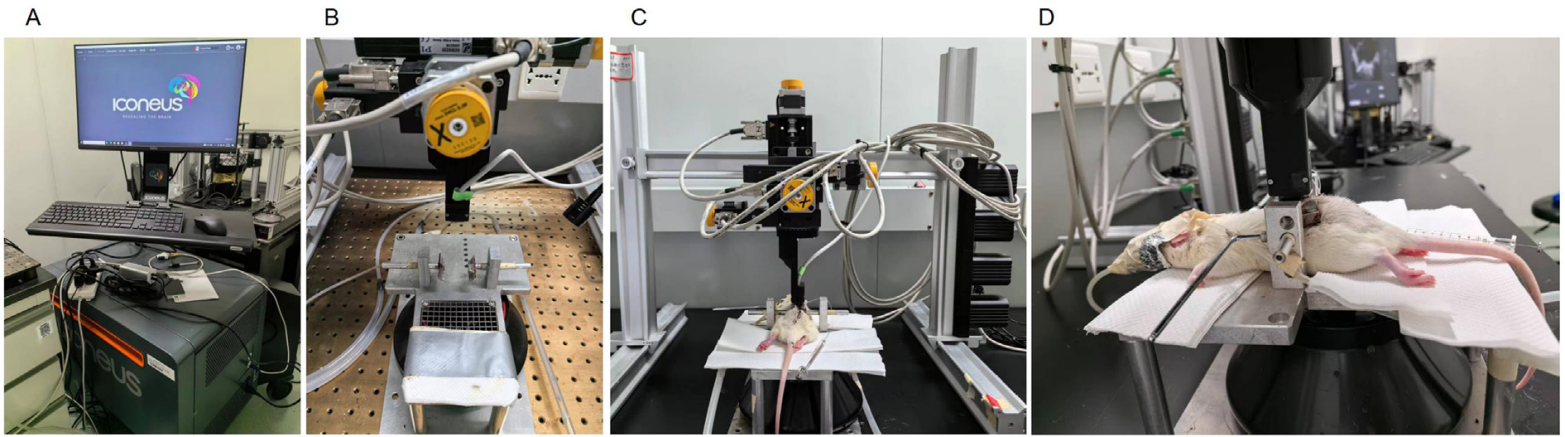
Apparatus for spinal blood volume testing. A.Ultrasonic ultrafast scanner. B. Special fixed shelf. C. Ultrasound probe. D. The positioning of the ultrasound probe on the rat.

Following an additional 30-minute stabilization period, the large blood vessel in the center of the spinal cord served as a reference point. Considering the rats had undergone ligation surgery mainly on the left side, the ultrasound probe was positioned 0.6 mm to the left of the center for detection (shown in Fig. 2). Baseline data were recorded for 180 s before slowly injecting 10 microliters of cerebrospinal fluid-containing gabapentin. The catheter was then flushed with an additional 10 microliters of cerebrospinal fluid. Each rat was recorded for a total of 60 min. The sham operation group underwent the same surgical procedure and received an equivalent drug dose for comparison with single intrathecal.

### Recordings in acute pain models

For naïve rats, the spinal cord area was exposed, and the administration end of the subarachnoid catheter was secured at the neck before individual housing. In the formalin-induced acute pain model, rats received an injection of cerebrospinal fluid containing gabapentin or control cerebrospinal fluid 30 min before ultrasound recording. After this period, the rats were anesthetized again, and the spinal cord tissue was exposed and allowed to stabilize for 30 min before commencing recordings. Baseline data were recorded for 180 s, followed by 50 µL 5 % formalin injection into the left hind paw. The subsequent 45 min were recorded in their entirety to capture the acute pain response.

### Gabapentin remarkably alleviated pain by regulating spinal vascular dynamics

**Fig. 3:**
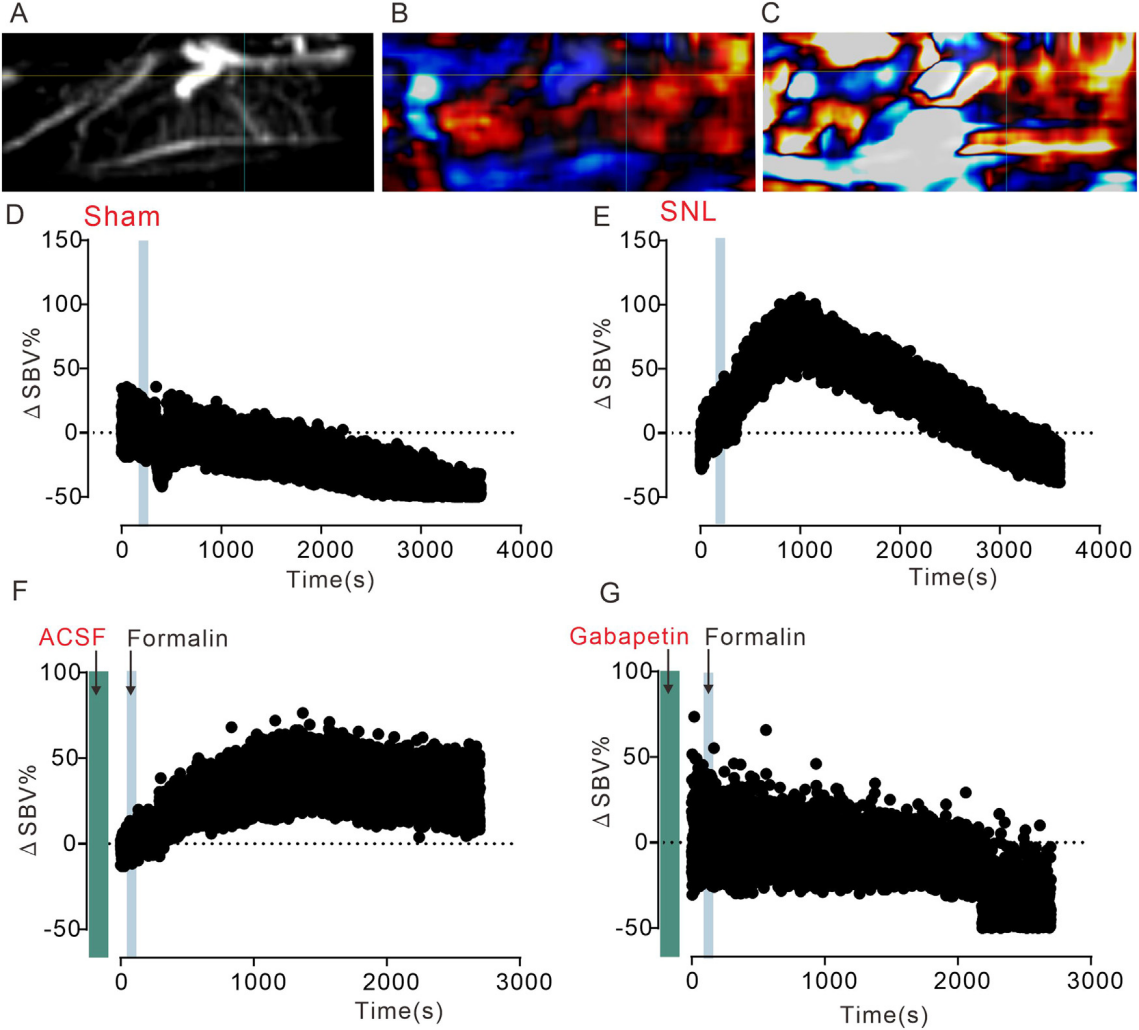
The hemodynamic response of major veins in the spinal cord to gabapentin injection in neuropathic and inflammatory pain. A. The major veins in the spinal cord. B. The veins before gabapentin injection. C. The veins after gabapentin injection. D. The functional spinal response to single intrathecal gabapentin injection in sham rats. E. The functional spinal response to single intrathecal gabapentin injection in neuropathic rats. F. The functional spinal response to formalin-induced acute pain when pretreatment of ACSF. G. The functional spinal response to formalin-induced acute pain when pretreatment of gabapentin.

Gabapentin, a widely used analgesic drug in clinical practice, demonstrates remarkable inhibitory effects on neuropathic and acute inflammatory pain. Our study investigated the hemodynamic changes in a rat model of neuropathic pain through ultrasound imaging (shown in Fig. 3A–C). Following the intrathecal injection of 100 µg gabapentin, we observed a significant increase in hemodynamic levels within the spinal cord glial layer (shown in Fig. 3E, the changes of average spinal blood volume, △SBV is 75.99 ± 51.92 %, Calculated the mean value within one minute at the peak as △SBV). This alteration persisted even after gabapentin administration. However, it's noteworthy that in the sham-operated group of rats, administering the same dose of gabapentin did not produce significant hemodynamic changes in the spinal cord glial layer (shown in Fig. 3D, △SBV is -57.42 ± 15.30 %). These observations suggest that spinal nerve injuries significantly disrupt the functional connectivity of spinal cord neurons in chronic neuropathic pain rat models. Furthermore, the injection of gabapentin effectively modulates neural activity in the spinal cord, thereby exerting its analgesic effects.

In contrast, we employed a distinct experimental approach for the formalin-induced acute inflammatory pain model. Before commencing ultrasound imaging, we intrathecally administered either 100 µg of gabapentin or control cerebrospinal fluid, 45 min before injecting 50 µL of formalin into the dorsal surface of the left hind paw. Our findings revealed that in the control group, the formalin injection significantly elevated hemodynamic levels within the spinal cord glial layer (shown in Fig. 3F, the changes of average △SBV is 47.21 ± 20.66 %). However, in the gabapentin-pretreated group, the formalin-induced hemodynamic changes were substantially blocked (shown in Fig. 3G, the changes of average △SBV is -18.90 ± 26.17 %). This indicates that formalin potently stimulates the spinal cord in the acute inflammatory pain model, eliciting intense pain signal transduction and marked hemodynamic shifts. Remarkably, pre-injection of gabapentin significantly curtailed these neuronal activations, ultimately leading to a considerable decrease in spinal cord hemodynamic levels.

In this study, we recorded the effects of gabapentin on spinal blood flow in rats with chronic neuropathic pain and on formalin-induced acute pain. This offers new insights into the neurovascular coupling mechanism, particularly regarding the mechanisms of analgesic drugs.

Current electrophysiological and optical imaging studies are constrained by a highly narrow spatial recording range, which undoubtedly poses certain obstacles to their widespread application. In contrast, neuroimaging techniques such as functional MRI exhibit significant advantages in the imaging field of view, capable of covering broader areas [9]. However, these techniques encounter challenges when dealing with small spinal cords in rodents, especially when assessing behavioral relationships in moving animals across different task scenarios. Additionally, motion artifacts caused by respiration pose a non-negligible problem. The combined effect of these factors results in significantly fewer neuroimaging applications in spinal cord research compared to brain studies. Therefore, there is an urgent need for imaging technology that combines high resolution, high sensitivity, and a large field of view to facilitate the in-depth development of spinal cord pain research.

Neurovascular coupling is a biological phenomenon that describes a physiological response process [10]. When neurons are activated, the small arteries in the brain region where they are located rapidly dilate, leading to a significant increase in blood flow [11]. This process precisely reflects hemodynamic changes in synaptic encapsulation areas, responding to changes in local neuronal activity. This exquisite coupling between nerves and blood vessels plays a pivotal role in various physiological states. Significantly during the transmission of peripheral signals from the dorsal root ganglion to the dorsal horn of the spinal cord, the blood vessels encapsulated by synapses change synergistically to ensure smooth nerve activity and accurate signal transduction. In a neuropathic pain model induced by spinal nerve injury, studies have found a significant enhancement in spinal glutamatergic transmission, which requires substantial ATP consumption. However, due to various factors such as mitochondrial damage, neurons need to resort to anaerobic glycolysis to barely maintain ATP supply. We observed that the analgesic drug gabapentin significantly regulates blood perfusion levels, which is crucial for correcting mitochondrial damage and synaptic plasticity. Furthermore, in acute pain, blood perfusion levels show a marked increase, consistent with the high ATP demand during acute pain episodes. When gabapentin was administered prophylactically, formalin-induced pain behaviors were reversed, indicating that this enhanced blood flow in acute pain was significantly blocked. Therefore, we have observed the phenomenon of analgesic drugs alleviating pain by regulating blood perfusion, which is essential for further elucidating the occurrence of pain and understanding analgesic mechanisms.

Why does vasodilation regulate ATP supply in neurons? Under physiological conditions, vascular endothelial cells are the primary cells that produce nitric oxide (NO) when blood vessels release NO. When vascular endothelial cells are stimulated, they activate nitric oxide synthase (NOS), which catalyzes the conversion of L-arginine into NO. NO is a potent vasodilator. It can diffuse to vascular smooth muscle cells, bind to heme within the cells, and activate guanylate cyclase (GC). GC catalyzes the conversion of guanosine triphosphate (GTP) into cyclic guanosine monophosphate (cGMP). As a second messenger, cGMP further activates protein kinase G (PKG), ultimately leading to the relaxation of vascular smooth muscle cells and vasodilation. At this point, glucose in the bloodstream can move into neurons due to the concentration gradient between the extracellular and intracellular environments, thereby increasing glucose levels and promoting ATP production. Therefore, there is a direct relationship between enhanced blood flow and ATP generation.

Consequently, by delving deeper into this neurovascular coupling phenomenon, we can better understand pain stimuli and the corresponding mechanisms of spinal cord hemodynamics, further unraveling the inherent mysteries of pain transmission. This aids in better comprehending the complex workings of the nervous system but also potentially offers new ideas and methods for pain management and treatment.

In present study, we selected gabapentin as the positive analgesic drug. On the one hand, we have conducted relevant animal studies in our previous research to evaluate the efficacy of gabapentin [5]. On the other hand, gender is also a crucial factor, as both clinical and animal studies have shown that gender does not appear to influence the analgesic effect of gabapentin. However, it should be noted that females generally perceive pain more accurately than males.

In conclusion, capitalizing on the favorable response elicited by the analgesic gabapentin, we have effectively harnessed spinal cord ultrasound imaging technology to provide meticulous insights into spinal cord hemodynamic shifts under both resting and task states within various pain models. This methodological approach assists in further elucidating the neural mechanisms that underlie pain perception, ultimately paving the way for developing more efficacious analgesic strategies.

## Limitations

None.

## Ethics statements

Animals in these experiments complied with the the ARRIVE guidelines and were carried out in accordance with National Institutes of Health guide for the care and use of laboratory animals (NIH Publications No. 8023, revised 1978). Due to the existence of gender differences in pain perception, this study only investigated male animals.

## CRediT author statement

**Yingzhuo Ding:** Conceptualization, Methodology, Writing - original draft. **Chunxia Yu and Qingqing Chi:** Validity tests, Data curation, Software. **Mengjiao Deng:** Visualization, Investigation. **Dongxia Duan and Jinbao Wei:** Supervision. **Yufei Xi and Qin Li:** Software, Validation. **Le Ma:** Writing- Reviewing and Editing.

## Declaration of competing interest

The authors declare that they have no known competing financial interests or personal relationships that could have appeared to influence the work reported in this paper.

## Data Availability

The raw data in our study are available from the corresponding author upon reasonable request.
